# Supplementary material for: Splice-Junction-Based Mapping of Alternative Isoforms in the Human Proteome
Source: Cell Rep. Author manuscript; Available in PMC 2020 Jan 15. (PMC6961840; doi:10.1016/j.celrep.2019.11.026)
Supplement: 2 [file NIHMS1546469-supplement-2.zip › DF1/PXD006675/RightVentricle/RightVentricle_32_TPM3_AISEELDHALNDMTSG.pdf]

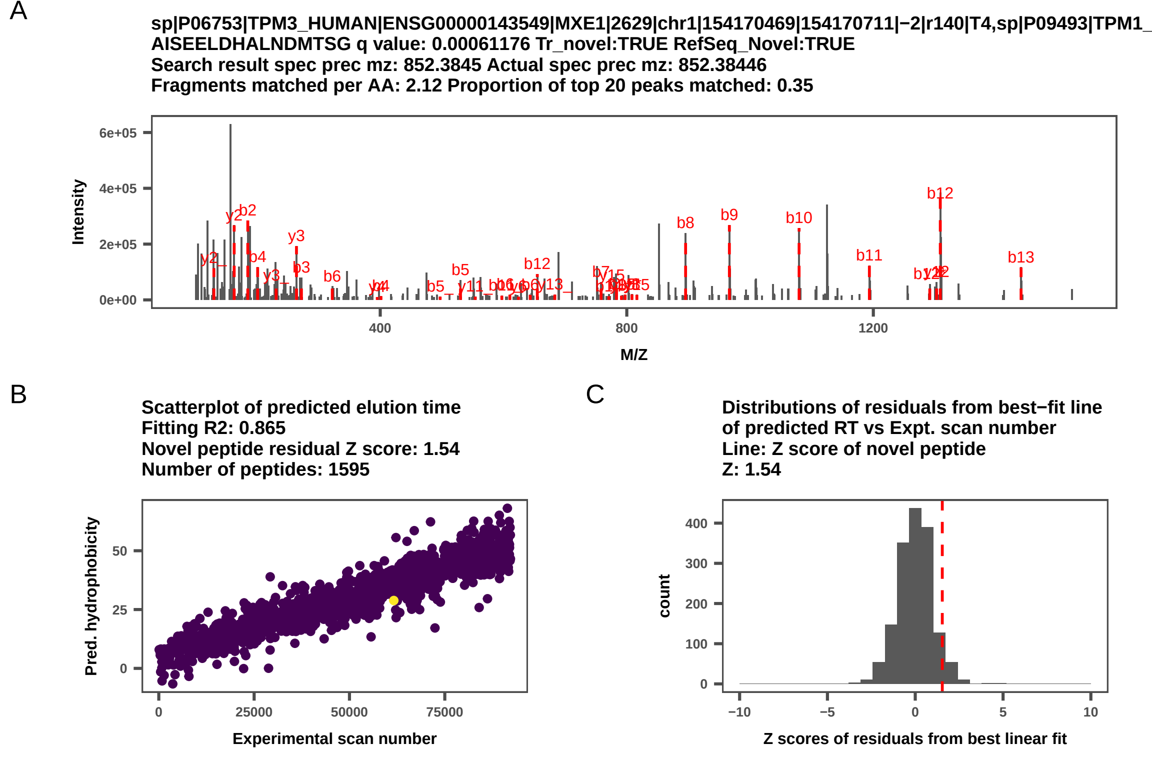
A
sp|P06753|TPM3_HUMAN|ENSG00000143549|MXE1|2629|chr1|154170469|154170711|−2|r140|T4,sp|P09493|TPM1_
AISEELDHALNDMTSG q value: 0.00061176 Tr_novel:TRUE RefSeq_Novel:TRUE
Search result spec prec mz: 852.3845 Actual spec prec mz: 852.38446
Fragments matched per AA: 2.12 Proportion of top 20 peaks matched: 0.35
Intensity
6e+05
4e+05
2e+05
0e+00
M/Z
400
800
1200
b2
y3
b4
b3
b6
b5
b12
b8
b9
b10
b11
b13
B
Scatterplot of predicted elution time
Fitting R2: 0.865
Novel peptide residual Z score: 1.54
Number of peptides: 1595
Pred. hydrophobicity
50
25
0
Experimental scan number
0
25000
50000
75000
C
Distributions of residuals from best−fit line
of predicted RT vs Expt. scan number
Line: Z score of novel peptide
Z: 1.54
count
400
300
200
100
0
Z scores of residuals from best linear fit
−10
−5
0
5
10
